# Editorial: Novel Strategies in Drug Development Against Multifactorial Diseases

**DOI:** 10.3389/fchem.2022.838063

**Published:** 2022-01-24

**Authors:** Cinzia Esposito, Catrine Johansson, Simone Di Micco

**Affiliations:** ^1^ Department of Molecular Life Sciences, University of Zurich, Zurich, Switzerland; ^2^ Oxford NIHR BRU, Botnar Research Centre, Oxford Centre for Translational Myeloma Research, Oxford University, Oxford, United Kingdom; ^3^ European Biomedical Research Institute of Salerno (EBRIS), Salerno, Italy

**Keywords:** multifactorial disease, drug discovery, drug resistance, biochemistry, drug repurposing

The complexity of multifactorial diseases, such as inflammation, cancer, neurodegenerative disorders, infectious pathologies represents a key obstacle in the success of therapeutic intervention. The occurrence of acquired drug resistance against molecular targeted therapies harnesses this biological complexity. In this context, multitargeting compounds can be a remarkable strategy for multifactorial disease treatment and to tackle drug resistance mechanisms. Indeed, in contrast to drugs that bind to a single target, the modulation of multiple macromolecules gives rise to additive and synergistic properties with the advantage of reduced side effects.

Despite the great potential of multitargeting drugs to treat multifactorial diseases, a limited number of these compounds have reached clinical trials or market. The selection of the right target combination and the design and identification of molecules endowed with a multi-bioactive profile, particularly challenging for unrelated targets, plays a key role in successful drug development. Thus, novel computational, chemical, biological, and biophysical approaches are urgently required for achieving new effective therapeutic tools towards the treatment of multifactorial diseases. This Research Topic gathers three research studies and one review.

Di Micco et al. adopted a drug repurposing strategy (Giordano et al., 2018a) to rapidly attenuate the current COVID-19 pandemic, demonstrating that the zonulin octapeptide inhibitor AT1001 (Larazotide acetate), currently in phase III trials in celiac disease (Troisi et al., 2021), binds the M^pro^ catalytic domain through molecular modeling and fluorescence resonance energy transfer (FRET) assay investigation. These findings combine with the well-demonstrated effect of AT1001 in improving mucosal permeability in ALI/ARDS, proposing it as a specific anti-SARS-CoV-2 multitargeting therapy for the global epidemic. This work led to the first generation of AT1001 derivatives, showing interesting anti-SARS-CoV-2 activity (Di Micco et al., 2021).

Recently, the development of an in silico/synthesis pipeline has been reported to identify new inhibitors of the glutathione-dependent enzyme mPGES-1, a valuable macromolecular target in both cancer therapy and inflammation therapy, has been reported (Di Micco et al., 2018). Specifically, the proposed approach was based on the virtual screening of commercially available fragments featuring aryl halide moieties, which represent the basic partners for Suzuki-Miyaura reactions (Miyaura and Suzuki, 1995), a very suitable synthetic strategy leading to platforms highly prone to further chemical modifications (Giordano et al., 2018b). Di Micco et al. implemented their in silico strategy, introducing phenyl and phenylethyl substituents as molecular probes mimicking boronic acid to improve aryl-bromide selection. The authors also enriched the input aryl bromide libraries to increase the chemical diversity exploration of molecular scaffolds to develop potential clinical candidates. In particular, the computer-aided approach could be potentially applied to design membrane-associated proteins in eicosanoid and glutathione (MAPEG) metabolism superfamily inhibitors, to target the prostaglandin pathway at multiple macromolecular levels for a more effective and safer therapy.

Zhang et al. applied bioinfomatic approaches on selected datasets acquired from the Gene Expression Omnibus (GEO) database to identify novel functional pathways, and diagnostic/prognostic biomarkers implicated in the pathogenesis of systemic juvenile idiopathic arthritis (sJIA), with as yet unmet medical need. The authors identified six hub genes and specifically suggested ARG1 and PGLYRP1 as potential biomarkers for the early diagnosis of sJIA. Furthermore, Zhang et al. revealed the contribution of the MAPK pathway and immune components such as platelets in the pathogenesis of sJIA paving the way for novel potential molecular targets for sJIA treatment.

Anthwal et al. surveyed the synthetic strategy to obtain 1, 3, 4 thiadiazole-based compounds endowed with different biological activities, such as anti-cancer, anti-viral, anti-diabetic properties. As the 1, 3, 4 thiadiazole could be considered a privileged structure (Di Micco et al., 2016), the authors suggested that it is suitable to develop anticonvulsant compounds.

The collected contributions provide different perspectives to develop multitarget compounds. This approach potentially represents an opportunity to obtain safer therapeutical treatment and to overcome drug resistance.

## References

[B1] Di Micco S. Musella S. Sala M. Scala M. C. Andrei G. Snoeck R. (2021). Peptide Derivatives of the Zonulin Inhibitor Larazotide (AT1001) as Potential Anti SARS-CoV-2: Molecular Modelling, Synthesis and Bioactivity Evaluation. Ijms 22, 9427. 10.3390/ijms22179427 34502335 PMC8431481

[B2] Di Micco S. Spatafora C. Cardullo N. Riccio R. Fischer K. Pergola C. (2016). 2,3-Dihydrobenzofuran Privileged Structures as New Bioinspired lead Compounds for the Design of mPGES-1 Inhibitors. Bioorg. Med. Chem. 24, 820–826. 10.1016/j.bmc.2016.01.002 26777299

[B3] Di Micco S. Terracciano S. Cantone V. Fischer K. Koeberle A. Foglia A. (2018). Discovery of New Potent Molecular Entities Able to Inhibit mPGES-1. Eur. J. Med. Chem. 143, 1419–1427. 10.1016/j.ejmech.2017.10.039 29133047

[B4] Giordano A. Forte G. Massimo L. Riccio R. Bifulco G. Di Micco S. (2018a). Discovery of New erbB4 Inhibitors: Repositioning an Orphan Chemical Library by Inverse Virtual Screening. Eur. J. Med. Chem. 152, 253–263. 10.1016/j.ejmech.2018.04.018 29730188

[B5] Giordano A. del Gaudio F. Johansson C. Riccio R. Oppermann U. Di Micco S. (2018b). Virtual Fragment Screening Identification of a Quinoline-5,8-Dicarboxylic Acid Derivative as a Selective JMJD3 Inhibitor. ChemMedChem 13, 1160–1164. 10.1002/cmdc.201800198 29633584 PMC6055880

[B6] Troisi J. Venutolo G. Terracciano C. Delli Carri M. Di Micco S. Landolfi A. (2021). The Therapeutic Use of the Zonulin Inhibitor AT-1001 (Larazotide) for a Variety of Acute and Chronic Inflammatory Diseases. Cmc 28, 5788–5807. 10.2174/0929867328666210104110053 33397225

